# Accuracy of clinical fetal weight estimation by Midwives

**DOI:** 10.1186/s12884-017-1242-7

**Published:** 2017-02-08

**Authors:** Assaad Kesrouani, Chady Atallah, Ramzi AbouJaoude, Norma Assaf, Hanaa Khaled, Elie Attieh

**Affiliations:** 1 Ob-Gyn Department, St Joseph University, Adib Ishac St, Achrafie, Beirut, Lebanon; 2 RM, Hotel-Dieu de France University Hospital, Beirut, Lebanon; 3 The Johns Hopkins Hospital, General Surgery, Baltimore, MD USA; 4 Johnston Willis Hospital, Obstetrics and Gynecology, Richmond, VA USA

**Keywords:** Estimation, Fetal weight, Clinical, Midwife

## Abstract

**Background:**

Clinical fetal weight estimation is a common practice in obstetrics. This study aims to evaluate the accuracy of fetal weight estimation by midwives, and to identify factors that may lead to overestimation or underestimation of fetal weight.

**Methods:**

A cohort prospective study in a Lebanese university hospital, included weight estimation of singleton pregnancies above 35 weeks. Multiple pregnancies, unclear dating, growth retardation, malformations and stillbirths cases are excluded. The estimated fetal weight is recorded by midwives in a sealed envelope and compared to true weight later. The effects of BMI, weight gain, parity, diabetes, hypertension, neonate’s sex and weight, uterine contractions, rupture of membranes and daytime or nighttime shift on these estimations were assessed.

**Results:**

One hundred and sixty-six patients were included. Mean birth weight was 3246 ± 362 g. Mean absolute percentage error of weight estimation was 8.5 ± 6.7% (0–30.9%). Estimation was within the correct range of ±10% in 63% of cases. Maternal and fetal factors did not significantly change weight estimation. Fetuses with birth weights more than 4000 tended to be underestimated by midwives. Estimation improved over time (nonsignificant).

**Conclusions:**

Maternal and fetal factors, except for macrosomia, have limited impact on estimation of fetal birth weight. Macrosomia is challenging because of a consistent tendency of underestimation by midwives.

## Background

Accurate estimation of fetal weight in late pregnancy provides valuable information for decision making in the management of birth, namely the mode and time of birth, as well as the subsequent management of the mother and the neonate [1]. This is especially true for fetuses at the extremes of birth weight, low birth weight and macrosomia [2]. Neonates with weights below the third percentile have a higher risk than the general population for developing neonatal respiratory distress syndrome, intraventricular hemorrhage and necrotizing enterocolitis during the perinatal period [3]. On the other hand, fetal macrosomia is associated with more maternal and fetal complications at the time of birth than other neonatal weight groups [4]. Maternal complications include increased cesarean rate, higher risk of injury to the genital tract, uterine rupture, as well as post-partum hemorrhage [5], while fetal complications include a higher risk for shoulder dystocia that could lead to bone fractures and permanent damage to the brachial plexus [1, 6–8]. The perinatal mortality rate and the rate of admission to the pediatric intensive care are also higher for neonates with macrosomia [5]. Correlation between estimated and true weight is variable among studies [9].

The purpose of this study is to evaluate the accuracy of the estimated fetal weight performed during labor by midwives at the Obstetrics and Gynecology department of a University Hospital in Beirut and to evaluate maternal and fetal factors that might affect the variation of this estimation.

## Methods

A prospective cohort study data collection included 166 parturients who delivered at Hotel-Dieu de France University Hospital, Beirut, from November 2010 to March 2011. Both last menstrual period and ultrasound were used to set the correct dating of pregnancies. The inclusion criteria were: a viable fetus of above 35 weeks, a singleton pregnancy and admission for labor and birth. Exclusion criteria consisted of the following: cases complicated by growth retardation, pregnancies with an unclear expected delivery date, multiple pregnancies, congenital malformations of the fetus, and stillbirths. The study is approved and registered by our institution’s review board under the number CEHDF-919.

Oral consent of participants was mandatory after explaining the potential scientific benefits and the absence of harm to the mother and her baby. A clinical estimation of the fetal weight by bimanual abdominal palpation (Leopold-Pavlik maneuver) was performed the midwife who was on duty (*n* = 4). Weight estimation was done when the patient was admitted to the hospital for delivery, with or without labor. The four midwives involved in this study are considered experienced and of equal obstetrical skills for initial evaluation of parturients as they have spent at least 2 years in the birth ward.

The result was dated and placed in a sealed envelope that was put in a closed box. At the end of the study, the box was opened for data retrieval and analysis. In order to avoid biased results, these estimations were made individually; the midwife did not have access to the patient’s chart or the prenatal records prior to weight estimation and no information was disclosed regarding the last estimated fetal weight by ultrasound they had at the doctor’s office.

The gold standard for comparing estimated fetal weight was the real birth weight, which was obtained immediately after birth using a digital scale. Analysis of the accuracy of fetal weight estimation was performed using the percent error {(estimated weight—actual weight)* 100/actual weight}. However, this formula has an inherent limitation because positive deviations from the real weight cancel the negative ones leading to underestimation of the magnitude of deviation from the real birth weight. To remedy for this bias, the absolute value of the percent error was taken as a measure of accuracy in addition to the percent error.

The percent of accurate weight estimation was reported; estimation was considered accurate if its percent error fell within a range of ± 10%. The validity of fetal estimation was assessed using intraclass correlation with absolute agreement.

To assess the effect of maternal and fetal factors on fetal weight estimation, percent of accurate estimation was compared using the Chi-Square test for categorical variables (rupture of membrane, presence of contractions, hypertension and otters). The Fisher’s exact test was also used for categorical variables when the expected cell count was less than five. The test of means (t-test) was used for continuous variables such as gestational age, maternal weight gain, maternal body mass index (BMI) and others. Data analysis was performed using SPSS version 18.

An Intraclass correlation coefficient of 0.75 was set as the minimum required value to have an agreement between the true and estimated method of fetal weight. Accordingly, a minimum sample size of 166 patients was required to get detect with a power of 80% an Intraclass correlation of 0.8 with a 0.1 confidence interval width. Alpha level was set at 5%. The study ended upon reaching the minimum sample required.

## Results

This study included 166 women. Population demographics is shown in Table 1. The mean ± standard deviation for body mass index, maternal weight gain, birth weight and gestational age was 28.03 ± 4.39 kg/m^2^, 12.9 ± 4.3 kg, 3246 ± 362 g, and 38.70 ± 1.21 weeks respectively. Boys and girls were equally distributed across the sample (84 girls and 82 boys) and 53% were night deliveries. Some patients were admitted for contractions that were felt by the patient but were not detected by the monitoring, along with cervical modification. Estimated fetal weights that were considered as correct (within the range of 10%) represent 63% of the sample.

**Table 1:** Demographics and clinical characteristics of women giving birth at a University Hospital in Beirut November 2010–March 2011

	N	%
Hypertension		
Hypertensive	3	1.8%
Not Hypertensive	163	98.2%
Rupture of membrane		
Membrane ruptured	28	16.9%
Membrane not ruptured	138	83.1%
Presence of contractions		
Contractions present	68	41.0%
Contractions absent	98	59.0%
Gender of neonate		
Female	84	50.6%
Male	82	49.4%
Shift of delivery		
Night	88	53.0%
Day	88	47.0%
Accuracy of weight estimation		
Correct estimation ^a^	105	63.3%
Missed estimation	61	36.7%
	Mean (SD)	Range
Body Mass Index	28.03 (4.39)	19.6–46.3
Maternal Weight Gain (Kg)	12.93 (4.30)	4–35
Birth Weight (gm)	3246 (362)	2280–4406
Gestational Age	38.70 (1.21)	35.2–42

^a^ Correct estimations of fetal weight are defined as estimation with percent error within an interval of 10%

Table 2 shows the various types of measures of difference between the estimated fetal weight and the real birth weight. The mean percent error was −1.8% with a standard deviation of 10.7% while the mean weight difference was −84.3 g with a standard deviation of 353.7 g. The negative sign of the mean percent error and the mean weight difference indicates a certain degree of weight underestimation by the midwives in general. This finding is more evident in Table 3 that describes the percentiles distribution of the percent error and weight differences. The median percent error was −2.6% and the median weight difference between estimated and real birth weight was −85 g, which means that more than half of the fetuses had their birth weight underestimated. Table 4 shows that estimations performed by the midwives had less than desirable agreement with the real birth weight; the Intraclass correlation coefficient was 0.573 with a 95% confidence interval of 0.42–0.69.

**Table 2:** Means, standard deviation and range of weight difference, absolute weight difference, percent error and absolute percent error for weight estimates performed by midwives at a University Hospital in Beirut

	Mean (SD)	Range
Weight difference (gm)	−84.3 (353.7)	−1110–850
Absolute weight difference (gm)	280.4 (230.5)	0–1110
Percent error	−1.8 (10.7)	−28–30.9
Absolute percent error	8.5 (6.7)	0–30.9

Weight difference is defined as estimated weight—Birth weight, Absolute weight difference is the absolute value of the weight difference. Percentage error is defined as (Estimated weight—Birth weight)*100/Birth Weight. Absolute percent error is the absolute value of the percent error

**Table 3:** Percentiles of weight difference, absolute weight difference, parcentage error and absolute percentage error for weight estimates performed by midwives at a University Hospital in Beirut

	Percentiles						
	5th	10th	25th	50th	75th	90th	95th
Weight difference (gm)	−673	−533	−330	−85	151	353	506
Absolute weight difference	13.5	38.8	100	222.5	390	593	720
Percent error	−17.9%	−14.3%	−9.6%	−2.6%	5.1%	12.8%	18.0%
Absolute percent error	0.4%	1.2%	3.1%	7.1%	12.7%	17.9%	22.5%

Weight difference is defined as estimated weight—Birth weight, Absolute weight difference is the absolute value of the weight difference. Percent error is defined as (Estimated weight—Birth weight)*100/Birth Weight. Absolute percent error is the absolute value of the percent error

**Table 4:** Intraclass correlation coefficients to assess validity of estimated fetal weight by midwives compared to fetal birth weight

Intraclass correlation coeficient	95% Confidence interval	
	Lower	Upper
0.573	0.419	0.686

**Fig. 1:**
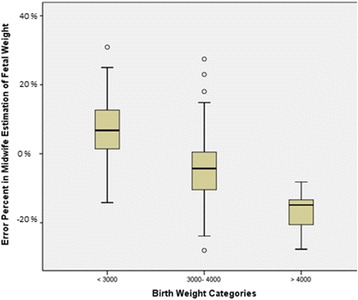
Box plot of quartiles of percent error in fetal weight estimation. The *bold horizontal lines* inside the boxes represent the medians. The lower and upper edge of the boxes represent the 25^th^ and 75^th^ percentiles respectively

Table 5 shows that the majority of maternal and fetal factors did not affect the accuracy of fetal weight estimation. The only factor involved was the birth weight group where all fetuses weighting more than 4000 g except one did not have correct weight estimation. Additionally, Fig. 1 shows that the birth weights of this group of neonates were mostly underestimated with a percent error less than minus10 %. There was however few babies in this sub – group and this would probably limit the interpretation of results.

**Table 5:** Effect of demographics and clinical factors on the accuracy of fetal weight estimation

	Correct estimation		Missed estimation		
	N	%	N	%	*P*- Value
Rupture of membrane					
Membrane ruptured	19	67.9%	9	32.1%	0.58
Membrane not ruptured	86	62.3%	52	37.7%	
Gender of Neonate					
Female	58	69.0%	26	31.0%	
Male	47	57.3%	35	42.7%	0.12
Presence of contractions					
Contractions present	45	66.2%	23	33.8%	0.52
Contractions absent	60	61.2%	38	38.8%	
Shift of delivery					
Night	58	65.9%	30	34.1%	
Day	47	60.3%	31	39.7%	0.45
Diabetes					
Diabetic	10	66.7%	5	33.3%	0.77
Not Diabetic	95	62.9%	56	37.1%	
Hypertension					
Hypertensive	2	66.7%	1	33.3%	0.70
Not Hypertensive	103	63.2%	60	36.8%	
Birth weight group					
< 3000	26	63.4%	15	36.6%	
3000–4000	78	65.5%	41	34.5%	
> 4000	1	16.7%	5	83.3%	0.053
Body Mass Index: Mean (SD)	28.04 (4.82)		28.02 (3.6)		0.972
Maternal Weight Gain: Mean (SD)	12.65 (4.59)		13.42 (3.75)		0.269
Parity: Mean (SD)	0.50 (0.70)		0.62 (0.78)		0.314
Gestational Age: Mean (SD)	38.66 (1.24)		38.79 (1.18)		0.529

Effect is measured as % difference in correct estimation across categories of nominal variables and as means difference for numeric continuous variables. A correct estimation is defined as a fetal weight estimation that falls within a 10% interval from the real birth weight

In the span of 6 months, systematic error decreased from about 9% during the first 4 months to about 6% during the last 2 months. However, statistical analysis of these data could not demonstrate a significant difference (*p* = 0.496).

## Discussion

Midwives represent key players in obstetrics. They sometimes have to rely on manual palpation for weight estimation and this certainly contributes to the subsequent management of labor. Sonographic estimation of fetal weight has also been widely used with a significant correlation between the neonate’s estimated and actual weight [10–12]. Nevertheless, fetal weight estimation based on palpation and clinical data is still a valid and reliable method [13–15]. Based on recent studies, clinical methods of fetal weight estimation compared favorably with sonographic estimation [13–16].

Clinical estimation of fetal weight correlated with birth weight, with an average margin of error around 8.6%. The percentage of cases in which the estimated weight is within the range of ± 10% of the actual weight is around 63%. These results are consistent with the results of several studies that found margins of error between 9 and 10%, and percentages of estimated weight contained in a range of ± 10% between 49 and 71%, depending on the fetal weight [13–17].

All patients in the study were at least at 35 weeks on the day of admission, aiming to have a homogeneous group. The time interval between clinical estimation and birth was very short for the 166 patients, which limited the effect of fetal growth on the difference between the estimated and the actual birth weight. The simplest estimation technique was the bimanual abdominal palpation (Leopold-Pavlik method). The accuracy of this clinical estimation is highly variable according to different authors and requires a certain degree of expertise [14, 17, 18], but it remains comparable to that of a sonographic estimation of fetal weight. Sherman et al. showed that the clinical estimation is more accurate than ultrasound in a group of birth weight between 2500 and 4000 g [13]. Another report confirmed this finding but only for babies less than 2500 g and between 2500 and 4000 g [16]. In addition, Hendrix et al. have demonstrated a higher accuracy of the clinical method, whatever the fetal weight [17]. Our study showed a significant correlation between the accuracy of the clinical method and the fetal weight at birth, with a systematic error which increases with fetal weight; it almost reached statistical significance (*p* = 0.053) despite the small sample size. However, the weight of macrosomic fetuses tended to be underestimated [9].

Few authors have investigated the factors that could interfere with the clinical estimation of term fetal weight. Fox et al. have shown that a maternal BMI greater than or equal to 30 significantly reduces the percentage of cases in which the estimated weight is within the range of ± 10% of the actual weight [19]. This finding was not confirmed in our study. Ben-Aroya et al. have shown that there is a statistically significant difference between clinical weight estimations depending on the daytime or nighttime shift but we failed to find such a difference [20]. The accuracy of the clinical estimations was demonstrated to be affected only by the neonate’s birth weight where the systematic error was negative, which means that the higher the fetal weight, the more it is underestimated. This could raise some questions about the usefulness of such an estimation, as macrosomia would be theoretically the desired indication for fetal weight estimation before birth. No relationship of the accuracy of the clinical method with the other factors has been found. Fundal height was reported as useful to estimate fetal weight and predict dystocia in term patients in labor [21].

In a study by Levin et al. experience didn’t seem to have an impact on clinical fetal weight estimation [22]. However, sharpening the skills of midwives in fetal weight estimation by the clinical method seems to be one of the key elements in the training of midwives. Evolution over time of the accuracy of estimations of fetal weight represents a new aspect of this subject that has been scarcely evaluated in other studies. This is an important variable that could have some impact on training programs for residents and midwives. Despite the fact that the margin of error has a tendency to decrease, change over time is considered non-significant; a larger number of patients may help in reaching a statistically significant increase in estimation accuracy over time. The introduction of a log book of clinical estimation of fetal weight could be interesting to include in a teaching program. Ultrasound estimation by midwives seems to be promising [23].

The major limitation of this study is the small number of enrolled patients thus reducing the strength of the study. Another limitation is the fact that the study did not assess the potential benefit that could be given to the patient in response to this clinical estimation, such as a decrease in fetal or maternal morbidity and mortality. A subsequent study addressing these two issues would possibly have a great impact on the recommendations for the obstetrical management.

## Conclusions

Most of the maternal and fetal factors did not affect the accuracy of fetal weight estimation, except for macrosomia where midwives tended to underestimate the weight. Improvement of the estimation over time is seen after 4 months but was not statistically significant. Larger trials are needed to evaluate how to improve this estimation in university teaching programs. This could also have an impact on rural medical centers with limited resources, as well as on parturients with limited prenatal care.

## Synopsis

Macrosomia influences prenatal fetal weight estimation.

## Abbreviation

BMI: Body mass index
